# Strengths and weaknesses of pneumococcal conjugate vaccines

**DOI:** 10.1007/s10719-023-10100-3

**Published:** 2023-01-18

**Authors:** Francesca Micoli, Maria Rosaria Romano, Filippo Carboni, Roberto Adamo, Francesco Berti

**Affiliations:** 1 GSK Vaccines Institute for Global Health, Siena, Italy; 2 GSK Vaccines, Siena, Italy

**Keywords:** Bacterial infection, *Streptococcus pneumoniae*, Polysaccharide, Conjugate, Vaccine

## Abstract

Multivalent vaccines addressing an increasing number of *Streptococcus pneumoniae* types (7-, 10-, 13-, 15-, 20-valent) have been licensed over the last 22 years. The use of polysaccharide-protein conjugate vaccines has been pivotal in reducing the incidence of invasive pneumococcal disease despite the emergence of non-vaccine serotypes. Notwithstanding its undoubtable success, some weaknesses have called for continuous improvement of pneumococcal vaccination. For instance, despite their inclusion in pneumococcal conjugate vaccines, there are challenges associated with some serotypes. In particular, *Streptococcus pneumoniae* type 3 remains a major cause of invasive pneumococcal disease in several countries.

Here a deep revision of the strengths and weaknesses of the licensed pneumococcal conjugate vaccines and other vaccine candidates currently in clinical development is reported.

## Introduction

*Streptococcus pneumoniae* (Spn) is an important human pathogen, well recognized as the etiological agent of community-acquired pneumonia (CAP), otitis media, septicemia and meningitis (together referred to as invasive pneumococcal disease [IPD]). Spn remains a leading cause of significant morbidity and mortality worldwide, with 5% of mortality due to pneumococcal infections in children under five years old. Pneumococci are common inhabitants of the respiratory tract and colonize the nasopharynx of 5–90% of healthy people (20–60% of school-age children and 5–10% of adults) [1–4].

Spn produces several virulence factors that are involved in the disease process. Among them the polysaccharide capsule is the most important virulence factor needed to invade the host and cause disease. The capsule is essential for the survival of the pneumococcus by protecting the organism from complement and subsequent killing of phagocytes.

Spn synthesizes > 100 types of capsular polysaccharides (CPSs), classified according to the unique glycan components and linkages that constitute each serotype. Among these, there are 23 serotypes responsible for 80–90% of invasive pneumococcal infections worldwide [5, 6].

Pneumococcal polysaccharide vaccines (PPVs) and pneumococcal conjugate vaccines (PCVs) targeting multiple Spn serotypes have been developed over the last years and are widely available for the prevention of pneumococcal disease. Since the 1970s, several PPVs have been developed containing capsular polysaccharides from 6 to 23 serotypes. Currently, only the 23-valent PPV is licensed in many countries worldwide, recommended for individuals aged 65 years and above as well as individuals aged 2 to 64 who had comorbidity, such as chronic cardiovascular disease and diabetes. Effectiveness of PPV23 against IPD in immunocompetent adults generally ranges from 56-to-81% but is lower in immunocompromised individuals. The vaccine is able to reduce the severity of CAP but not to prevent it and does not reduce the incidence of non-invasive pneumonia. Furthermore, PPV23 is not effective in children < 2 years of age, does not generate immunological memory and does not lead to reduced carriage [7, 8]. To improve the effectiveness of the vaccine in children, PCVs were developed, in which capsular polysaccharides are conjugated to a carrier protein, overcoming most of the limitations of PPVs. To date PCV10, PCV13, PCV15 and PCV20 are available on the market (Table 1) [9].

According to the WHO report [10], Spn is among the four priority pathogens for antimicrobial resistance (AMR) for which licensed vaccines already exist and for which increasing coverage of licensed vaccines is recommended, in line with WHO immunization targets to maximize the impact on AMR.

## Licensed conjugate vaccines: toward higher valency

In order to improve vaccine effectiveness in children, several PCVs (Table 1) have been licensed. A 7-valent pneumococcal conjugate vaccine (Prevnar, Pfizer) was developed and licensed in the United States and European Union in 2000 and 2001, respectively. PCV7 comprises CPS purified from the seven most common IPD serotypes in the United States and conjugated with a genetically detoxified mutant of diphtheria toxoid, CRM_197_ (Table 1). The use of PCV7 resulted in a rapid reduction of IPD cases in young children and a sharp decline in pneumococcal infections in adults [11]. In the USA, the incidence of IPD in 2004, i.e. after introduction of the vaccine, was reduced by 77% in children < 1 year of age, by 83% in children at 1 year and by 73% in 2-year-old children [12]. A decrease in non-invasive pneumococcal infections such as acute otitis media (− 20%) was also found in the US population [13].

Due to the selection pressure exerted by PCV7, other serotypes not included in the vaccine became problematic, such as serotype 19A [14, 15]. This process, known as serotype replacement, drove the development of PCV10 (Synflorix, GSK) and PCV13 (Prevnar 13, Pfizer) [16, 17], that have been available since 2009 [18]. These vaccines contain the 7 pneumococcal capsular polysaccharide serotypes in PCV7, plus 3 or 6 additional serotypes (Table 1) selected based on the evolving worldwide epidemiology of pneumococcal disease. Another 10-valent PCV vaccine, (Pneumosil, Serum Institute of India), with overlapping serotypes with PCV10 and PCV13, was prequalified by WHO in 2019.

In general, PCV10 and PCV13 reduced nasal colonization of vaccine types and led to herd immunity in the population [19–22]. Although the main selection criterion for serotypes in PCV10 and PCV13 was based on disease burden, serotypes associated with antibiotic resistance are also included [23] and several studies suggest that current PCVs have been highly effective in reducing the prevalence of drug-resistant infections [24, 25]. Five years after the first PCV use in the USA, IPD caused by multidrug-resistant (MDR) strains decreased by 84% in children under 2 years and penicillin-resistant IPD in adults over 65 decreased by 49% [26]. In South Africa, the use of PCV led to an 82% reduction in the rate of penicillin-resistant pneumococcal disease in children, and a 47% reduction in disease caused by penicillin-susceptible strains [27].

To further improve coverage, FDA recently approved PCV15 and PCV20 from Merck and Pfizer respectively [28–30], containing 15 or 20 different serotypes, that have shown an impact in reducing noninvasive illnesses such as acute otitis media, nonbacteremic pneumonia, and sinusitis [31]. However, unless a tipping point is reached in the coverage such to prevent serotype replacement, the efficacy of these vaccines will slowly wane. Even if this is technically feasible, manufacturing costs of the vaccine would render the development challenging particularly in developing countries. A reasonable solution would be to continously vary the formulation based on the most relevant circulating IPD serotypes. In silico simulations suggest that adapting the vaccine composition based on the latest epidemiological data holds the promise to reduce IPD burden, however the vaccine woud not be able to completely eradicate pneumococcal colonization in the population [32].

All licensed conjugates use CRM_197_ as carrier protein, except Synflorix that has Protein D for serotypes 1, 5, 6B, 7F, 9 V, 14, 23F and 4, TT for serotype 18C and DT for serotype 19F. Recently PCV13 with TT as carrier protein has been licensed by Walvax (Products list walvax.com).

**Table 1:**
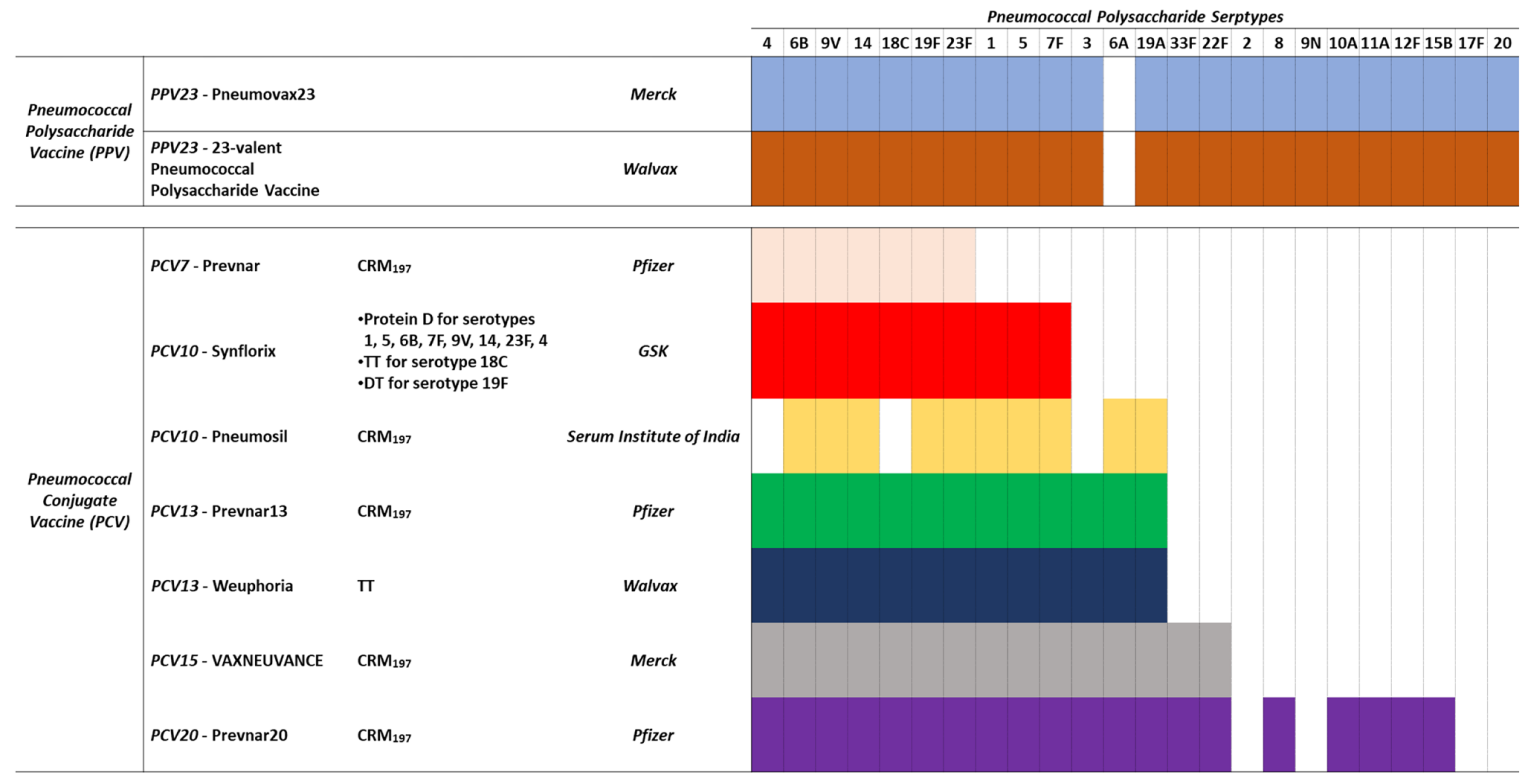
Licensed pneumococcal polysaccharide vaccines (PPVs) and pneumococcal conjugate vaccines (PCVs) targeting multiple Spn serotypes

## Inconsistent vaccine efficacy

Conjugate vaccines successfully protect from infection, but not all pneumococcal serotypes are covered with equal potency. For some serotypes, levels of the functional anti-CPS antibodies elicited by licensed vaccines and detected by opsonophagocytic killing (OPK) of bacteria, are not ideal. Among these there are serotypes 1, 3 and 5 [33–36] that we will further comment in the following paragraphs.

### Type 1

It is generally accepted that negatively charged and neutral polysaccharides are type 2 T cell-independent antigens (TI-2, or thymus-independent type 2 antigens). This includes most of the pneumococcal CPS [37, 38]. TI-2 polysaccharides are unable to recruit CD4 + T-cell help because they do not interact with the major histocompatibility complex class II (MHC-II) molecules [39].

**Fig. 1:**
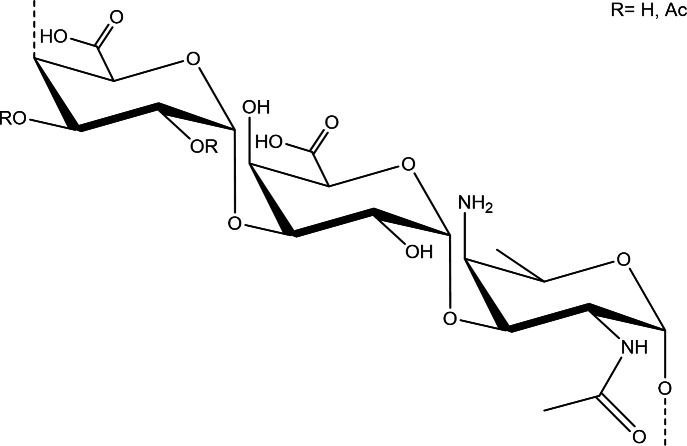
Schematic representation *Streptococcus pneumoniae* serotype 1 capsular polysaccharide repeating unit →4)-α-D-Gal*p*A2/3Ac-(1→3)-α-D-Gal*p*A-(1→3)-D-AAT-α-Gal*p*-(1→.

However, certain types of glycans, specifically those with a dual or zwitterionic charge motif, can indeed activate T cells through the traditional MHCII pathway [40]. Type 1 *S. pneumoniae* polysaccharide capsule (Spn-1) (Fig. 1) is one of the most studied among these molecules. It has been shown that Spn-1, even without conjugation to a carrier protein, can be processed and presented by antigen-presenting cells utilizing the MHC-II pathway, which leads to Spn-1-induced T cell proliferation [41–43].

However, in a more recent study, it was shown that Spn-1 polysaccharide does not induce memory B cell formation in humans, similarly to other polysaccharides. Peripheral blood mononuclear cells obtained from adults enrolled in a randomized clinical trial to investigate memory B cell responses following immunization with the 23-valent pneumococcal plain polysaccharide vaccine were used. Administration of this serotype 1 containing vaccine resulted in the depletion of serotype 1 antigen-specific pre-existing memory B cells compared to baseline. This finding indicates that this zwitterionic polysaccharide is not processed by a classical T-dependent mechanism within the MHC-II pathway [44].

Also, Bjarnarson et al. showed that both zwitterionic (Spn-1) and non-zwitterionic (Spn-19F) polysaccharide caused hyporesponsiveness after booster in a neonatal murine model [45].

As opposed to the expected immunological activity even in absence of conjugation, marketed conjugate vaccines containing Spn-1 induce low levels of functional anti-CPS antibodies against this serotype [46, 47], a major cause of meningitis in sub-Saharan Africa [48] .

This is presumably due to the obscuring of protective epitopes during chemical activation and conjugation to carrier proteins. Spn-1 CPS harbors the unusual monosaccharide, 2-acetamido-4-amino-2,4,6-trideoxy-d-galactose (D-AAT), that contains a free amine (Fig. 1). Since the manufacturing of marketed conjugate vaccines uses reductive amination (Prevenar) or 1-cyano-4-dimethylaminopyridine activation chemistry (Synflorix, Pneumosil) that reacts with free amines [49], chemical derivatization of a certain fraction of the D-AAT moieties may lead to reduced vaccine efficacy [34]. In addition, polysaccharides are depolymerized during manufacturing, for instance by sodium periodate-mediated diol oxidation, which may also lead to an additional reduction of efficacy.

### Type 5

**Fig. 2:**
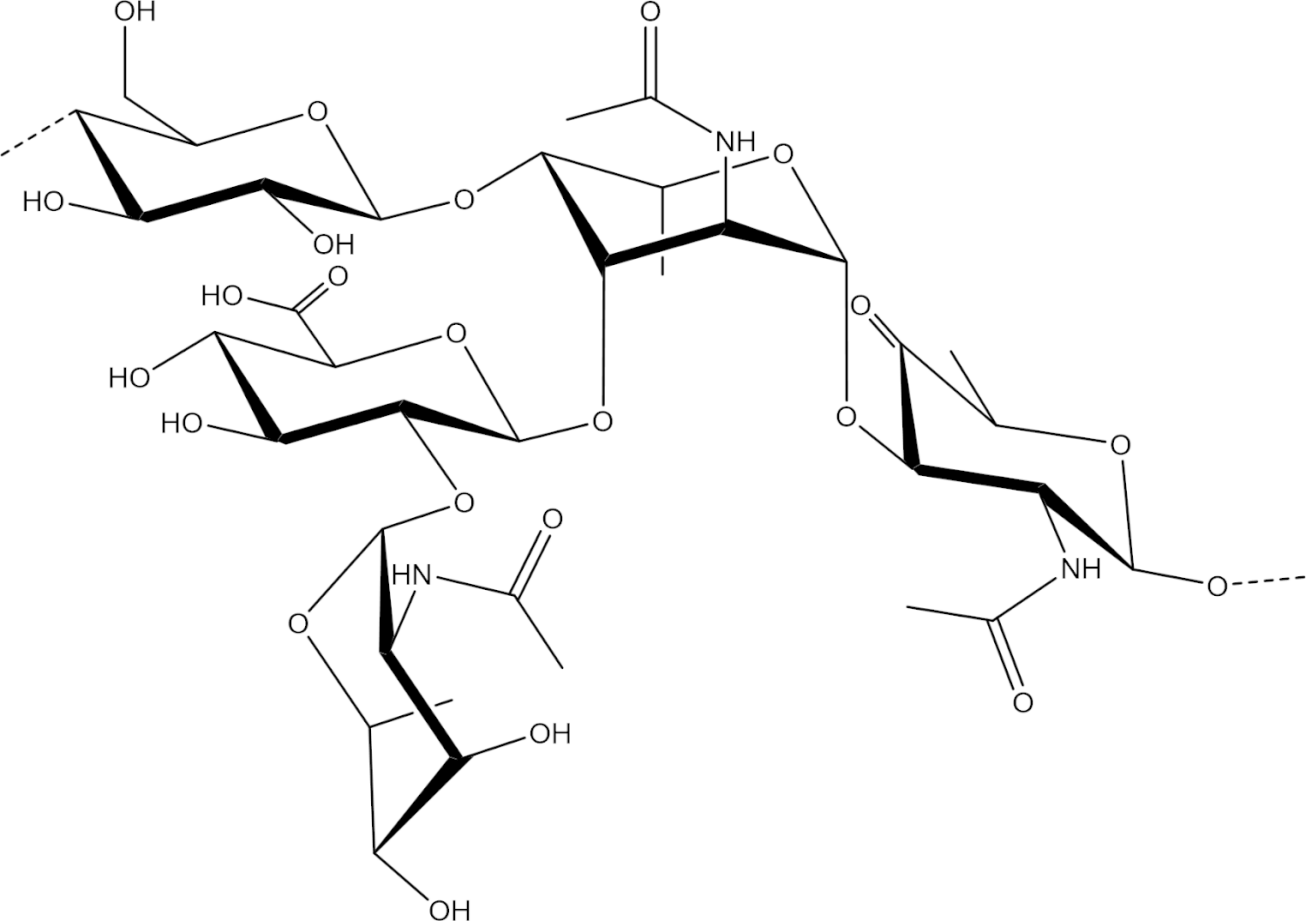
Schematic representation *Streptococcus pneumoniae* serotype 5 capsular polysaccharide repeating unit

*S. pneumoniae* serotype 5 (Spn-5) has been reported as the fifth most prevalent serotype causing invasive pneumococcal disease among young children globally [50]. Also, in Vancouver, Canada, a large cluster of IPD associated with this serotype (multilocus sequence type or MLST 289) was identified, resulting in a large outbreak of pneumococcal disease that spread throughout Western Canada from 2005 to 2009 [51]. Marketed conjugate vaccines are not fully efficacious in preventing Spn-5 infections and again the cause of this behavior is to be found in some stability problems following conjugation. The root causes of the instability of Spn-5 in the chemical conjugation process with the carrier protein can be easily identified looking at the repeating unit of the polysaccharide (Fig. 2). Serotype 5 repeating unit is composed of 5 different residues, 3 of which form the backbone of the polysaccharide and the remaining 2 are part of the branch attached to the central N-acetyl-L-fucosamine. Beyond D-glucose (part of the backbone) and D-glucuronic acid (part of the branch), two rare deoxyamino residues complete the structure: N-acetyl-L-pneumosamine (L-PneuNAc) and 2-acetamido-2,6-dideoxy-D-xylos-hexos-4-ulose (Sug*p*).

In particular, the lability of this latter ketoamine sugar, part of the backbone, is the main reason for the manufacturing problems of the conjugate vaccine. In fact, in the reductive amination reaction that allows the attachment of the polysaccharide to the carrier protein, the keto group of this sugar can be partially or totally reduced. Using conventional conditions for reductive amination, the vast majority of the Sug residue disappears in favor of three possible compounds: N-acetyl-D-quinovosamine (QuiNAc, from reduction of the ketone function to an alcohol function), N-acetyl-D-fucosamine (FucNAc, isomer of the N-acetyl-D-quinovosamine) and a third side product resulting from a more considerable conversion of the Sug residue. It was discovered that conversion of the Sug compound to this third side product was harmful to the immunogenicity of the polysaccharide, even when this conversion is only partial, while the QuiNAc or FucNAc conversion has no notable influence. Several attempts have been made to overcome this problem, in particular by modifying the conjugation conditions. A possible modification is to shift the pH of the reductive amination reaction from 8 to a more acidic pH (between 4 and 6.5) obtaining a degree of modification of the Sug residues decreased by at least 65% compared to the conventional method with most of the conversion to QuiNAc, with therefore less impact on the conjugate immunogenicity. Another possibility is to convert completely Sug to QuiNAc or FucNAc, reducing the ketone group with a strong reducing agent such as NaBH_4_. However, it is necessary to fragment the reduced polysaccharide to reintroduce the terminal aldehyde groups, while preventing any modification by subsequent reductive amination [52]. Seeberger and colleagues [33] synthesized a series of oligosaccharides resembling the Spn-5 repeating unit. In particular, a fragment containing a secondary alcohol in place of the labile ketone of Sug residue elicited much higher antibody titers than the multivalent Prevnar13 vaccine with significantly higher opsonophagocytic killing properties and a robust immunological memory. They also demonstrated with rabbit and human reference sera that the branch, and in particular PneuNAc residue, was mainly involved in antibody recognition and avidity, corroborating that the replacement of the non-essential, labile keto group of Sug did not affect immunogenicity, resulting in a promising more stable vaccine candidate.

### Type 3

Serotype 3 is a particular problem and merits discussion. PCVs have been pivotal in reducing the incidence of IPD despite the emergence of non-vaccine serotypes. However, the incidence of disease caused by Spn-3 isolates has not declined despite its inclusion in the 13-valent PCV vaccine and vaccine effectiveness has been reported as non-significant for this serotype [53, 54], leading to it being recorded as a non-vaccine type in some vaccine efficacy studies. Spn-3 produces profuse quantities of capsule. Unlike the vast majority of other serotypes, Spn-3 capsule is not covalently attached to the bacterial surface resulting in constant polysaccharide release [55].This could lead to less capacity of antibodies bound to the capsule at a distance from the bacterial surface to induce bacterial killing [55]. Furthermore, when compared to other serotypes associated with IPD, Spn-3 has a high case carrier ratio and children have been shown to carry high levels of antibody to serotype 3, presumed to be due to a high rate of natural exposure, but low duration of carriage, supporting the suggestion that this serotype is highly invasive.

**Fig. 3:**
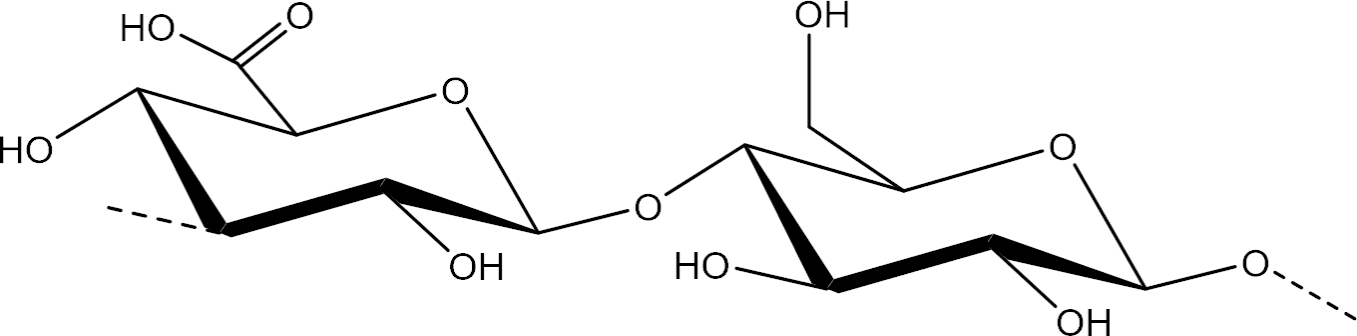
Schematic representation *Streptococcus pneumoniae* serotype 3 capsular polysaccharide repeating unit →3)-β-D-Glc*p*A-(1→4)-β-D-Glc*p*-(1→.

Furthermore, the chemical composition of this polysaccharide (Fig. 3) provides some specific features to it, such as a “wet” or mucoid appearance when grown on agar plates, which could imply a peculiar resistance to opsonic killing *in vivo*.

Choi et al. assessed the serotype specific protective effects of PCV13. It was shown that 0.2 µL of serotype 3 bacterial culture supernatant was sufficient to overwhelm the protective effect of the vaccine-induced antibody in passively immunized mice. It was estimated that eight times more antibody is needed to be generated by PCV in order to induce protection against serotype 3 invasive disease. Notably disease caused by Spn-3 is particularly severe including complications of pneumonia and adverse cardiac events [30].

### Lacking cross-protection

**Fig. 4:**
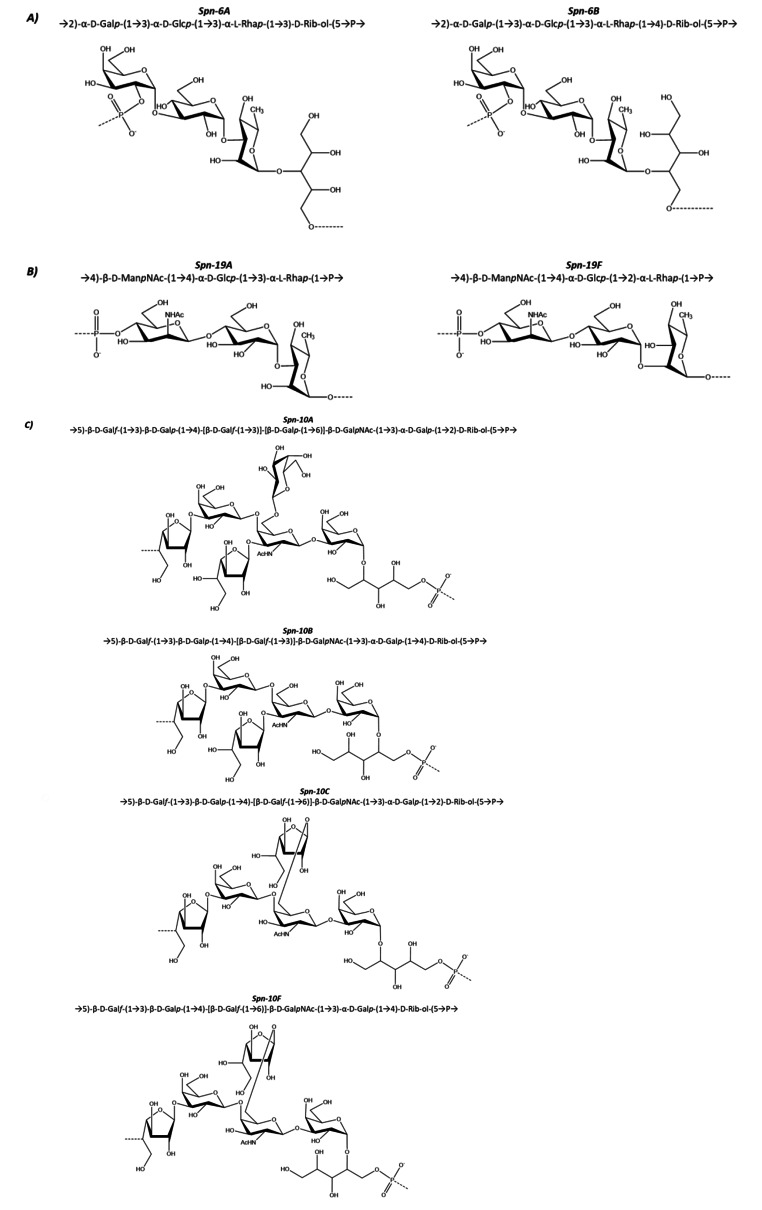
Schematic representation of *Streptococcus pneumoniae* serotypes sharing similar structure for which cross-protection should be expected: ***(A)*** Spn-6A and Spn-6B; ***(B)*** Spn-19A and Spn-19F; ***(C)*** Spn-10A, Spn-10B, Spn-10C and Spn-10F.

Given the large number of pneumococcal CPSs, understanding their cross-protection is an important element to define the best strategy for the formulation of pneumococcal vaccines and evaluate the vaccine efficacy. Indeed, among the various CPSs, there are serotypes that have a sufficiently similar structure for antibodies cross-protection, such as those of serotypes 6, 19, 10 (Fig. 4).

Among serotypes 6A, 6B, 6C and 6D, which differ slightly in their structure. Spn-6A and Spn-6B are those with the greatest clinical importance [56]. Spn-6B and Spn-6A polysaccharides (Fig. 4) are linear copolymers that differ from each other in a single binding and it has been shown that serotype 6B can elicit antibodies that cross-react with serotype 6A [57]. Both serotypes 6B and 6A are frequent causes of infections, but due to the improved hydrolytic stability of serotype 6B over 6A [58], early commercial pneumococcal vaccines contained only serotype 6B as the sole representative of serogroup 6. However, studies have shown that although vaccines containing serotype 6B elicit antibodies that cross-react with type 6A, not all anti-6B antibodies are functionally cross-reactive [57].

Over the past decade, the synthetic approach has been used to produce saccharide structures shared by several Spn serotypes [59]. Synthetic saccharide fragments of Spn-6B, conjugated with the keyhole limpet hemocyanin (KLH) protein, induced antibodies capable of cross-reacting to both serotype 6B and 6A in mice and rabbits, as well as stimulating phagocytosis of microbial cells of pneumococcus serotypes 6A and 6B [60].

Spn-19A and Spn-19F polysaccharides are another example of serotypes consisting of very similar repeating units, differing only in the position of a glycosidic bond (Fig. 4). However, the increased number of infections caused by serotype 19A after vaccination with pneumococcal vaccines containing only serotype 19F seems to indicate that there is not antibody cross-protection between the two serotypes [61], probably due to conformational differences between the two polysaccharide chains [62].

A few years ago, a chimeric antigen comprising a repetitive unit of Spn-19A and Spn-19F was synthesized and conjugated to CRM_197_. The conjugate, tested in rabbits, was able to induce high antibody titers able to recognize both native serotype 19A and 19F. Furthermore, the antibodies were able to kill both strains of pneumococci [63]. Other researchers have synthesized a small library of compounds containing different combinations of the common disaccharide ManNAc-β-(1→4)-Glc present in the repeating unit of Spn-19F and 19A, demonstrating with a microarray format that the disaccharide phosphorylated at the upstream end is strongly recognized by antibodies in reference sera of rabbits immunized against either Spn-19F or Spn-19A [64].

Serotype 10 includes five variants: 10A, 10B, 10C, 10F and the recently identified 10D [5]. Among them, Spn-10A has been included in the latest generation PCVs (Table 1) being the most prevalent. The Spn-10A, Spn-10B, Spn-10C and Spn-10F consist of a repeating unit with a backbone of galactofuranose, galactopyranose, N-acetylgalactosamine and ribitol-phosphate, differing mainly in the position of a glycosidic bond to the ribitol-phosphate and for side chain substitutions (Fig. 4) [3, 65, 66]. The new serotype 10D has instead a significantly different backbone compared to the others. Cross-reactivity between serotypes Spn-10A, Spn-10B, Spn-10C and Spn-10F is complex with unclear structural reasons, serotype-specific rabbit antisera generated against a serotype recognized other serotypes, albeit with less affinity [67]. Ravenscroft and Kuttel recently published a molecular modeling study that together with relevant serological studies supports the inclusion of serotype 10A in a vaccine to best protect against serotypes 10 disease [68].

## Impact of PCVs on otitis media

The success of capsule-based vaccines against invasive disease is unfortunately counterbalanced by weak efficacy at mucosal sites, such as for pneumonia and otitis media. Like many other successful bacterial pathogens, Spn undergoes phase variation, during which, surface components, such as capsule, shift stochastically from high to low levels of expression.

The frequency of specific phase-variants in distinct anatomical sites is the result of anatomical-site specific selective pressures in the host environment [39]. Bacteria on the mucosal surface have been shown to be less encapsulated than blood-isolated counterparts, thereby exposing surface adhesins, so that antibodies directed at capsule are at a disadvantage in promoting opsonophagocytosis at the mucosal level. In addition to this genetic adaptation to a niche, a newly recognized phenomenon of capsule shedding also decreases the efficacy of anti-capsular antibodies [40]. Upon entering the lung, Spn encounters antimicrobial peptides and as a defense, the bacteria shed most of their capsule over several hours, which could render capsule based vaccination less effective in these circumstances.

It has been observed also some serotype specificity in acute otitis media (AOM) infections. AOM is an important infectious disease of the respiratory tract among children and is a leading cause of physician visits and antibiotic prescriptions. Serotype 19A ST320 seems the predominant cause of pneumococcal mastoiditis, a suppurative complication of AOM, and this serotype causes persistent symptoms even when treated with high doses of β-lactam antibiotics.

In Taiwan, Spn-19A ST320 clone was highly prevalent under a low PCV7 vaccination rate. About 50% of cases of IPD among children aged < 5 years were caused by Spn-19A ST320. In a retrospective study conducted between 2009 and 2012 in AOM in patients aged 0–18 years by sampling middle-ear fluid, pneumococcal AOM was observed in 108 (24.8%) of 436 episodes. Serotype 19A ST320 (50, 58.8%) was the most frequent serotype associated with AOM.

In another study conducted in Spain, the epidemiology of pneumococcal OM was analyzed before and after PCV13 introduction in 2010. Between 2008 and 2016, the middle ear exudates from 2653 children under 14 years of age with OM were collected and Spn was isolated in 235 (8.9%) of cases. The 204 available isolates were serotyped and distributed in three 3-year periods: one before and two after PCV13 introduction. The study indicated that the introduction of PCV13 did change the epidemiology of pneumococcal OM, with a decrease in the rate of vaccine serotypes accompanied by an increase in the diversity of non-vaccine serotypes and the clonal spreading of different single clones in each region. Exceptions were observed such as the persistence of serotype 3 and a weak re-emergence of serotype 19F.

In a larger longitudinal observational study done in the US, involving healthy children seen as outpatients in a private pediatric practice, children aged up to 30 months who had received the full primary series of PCV13 were followed up to age 30–36 months to identify episodes of AOM, by collection of middle-ear fluid (MEF). AO MEF for the serotypes common to PCV7 and PCV13 (4, 6B, 9V, 14, 18C, 19 F, and 23F) and the six additional serotypes specific to PCV13 (1, 3, 5, 6A, 7F, and 19A) were assessed. The control group was deriving from another longitudinal study conducted from 2007 to 2009, where newborns had been vaccinated with PCV7, had MEF prospectively collected at the onset of AOM and had been followed up until age 30 months.

The study demonstrated the PCV13 effective in reducing AOM associated with Spn, especially episodes caused by the six serotypes additional to those in PCV7. An 86% relative reduction of the six additional serotypes in PCV13 in MEF samples obtained during AOM was achieved, with a 91% relative reduction for serotype 19A. No serotype 6A isolates were identified in the MEF samples from children who were vaccinated with PCV13. No decrease in the number of children with AOM caused by capsular serotype 3 was found, although the incidence of this serotype in both cohorts was low. Isolates from three of the six additional serotypes in PCV13, namely 1, 5, and 7F, were not detected in either cohort. Notably, in spite of its presence in both PCV7 and PCV13, the incidence of disease from serotype 19F is considerable, albeit at much lower frequencies than prior to PCV7 introduction.

Additional papers based on analysis of prescriptions for OM consolidate the finding that AOM disease burden remains high in children of all ages despite reductions were observed following the introduction of PCV7 and PCV13 [69].

Overall, these studies support partial impact of PCV vaccination in OM and highlight variability of efficaciousness depending on AOM etiology and circulating pneumococcal serotypes [70, 71].

## New technologies for making conjugate vaccines

Despite widespread utilization of PCVs and the consequent disease reduction, the research is still active and innovative technologies are being explored for the synthesis of higher valency polysaccharide-based vaccines, with increased efficacy, coverage and at reduced costs. Indeed, the development of PCVs containing additional serotypes remains a public health priority due to serotype replacement and the shift to non-vaccine containing serotypes.

Incorporating additional serotypes to existing PCVs has resulted in reduced immune responses to individual serotypes very likely due to carrier suppression [29, 72, 73].

A 24-valent PCV (VAX-24, Vaxcyte) based on the cell-free protein synthesis (CFPS) platform is being tested in a Phase 1/2 trial in healthy adults (NCT05266456). This conjugation approach proposes the use of eCRM carrier protein containing multiple non-native amino acids (nnAAs) located outside of the primary T-cell epitope regions, for site-specific covalent conjugation of the pneumococcal CPSs through click-chemistry. This results in a more consistent conjugation process and in increased polysaccharide:protein ratio, enabling the inclusion of more serotypes while minimizing carrier-mediated immunological interference. In a study in rabbits, VAX-24 showed comparable IgG responses and OPA titers to Prevnar13 and higher responses than PPV23 [69].

Complete or semisynthetic oligosaccharides of serotype 1, 2, 3, 5 and 8 capsules have been produced and demonstrated to be equivalent or superior to PCV13 in murine and rabbit models [33, 34, 74, 75]. Synthetic oligosaccharides can be designed to carry linkers for site-selective protein conjugation while keeping protective epitopes intact. Chemically synthesized conjugates can be used independently or as a supplement to the current PCV13 formulation [76].

Also, protein capsular matrix vaccines (PCMVs) were developed [77], avoiding introduction of unwanted modifications in the carbohydrate epitopes: PCMVs are prepared by mixing CPS, carrier proteins and chemical crosslinkers form aggregates where unmodified CPS is trapped with the protein. The immunogenicity of PCMVs is dependent on the particle size, suggesting that larger particles may be preferentially taken up by antigen presenting cells.

Outer Membrane Vesicles (OMV) have been also proposed for the display of Spn-14 glycans. OMV combine optimal size for immune stimulation with multiple antigens display and self-adjuvanting properties [77, 78]. Bacteria are engineered to display saccharide chains on OMV resulting in so called glyOMV, simplifying the manufacturing process that does not include any conjugation step. In mice, Spn-14 glyOMV induced IgG and opsonophagocytic activity comparable to those induced by PCV13 [79].

PCVs are unable to protect against non-vaccine serotypes and unencapsulated bacteria and other limitations include high costs due to complex formulations, low immune response for certain serotypes and several doses needed, making them less likely to be used extensively in developing countries where the need is the highest. Therefore, a pneumococcal vaccine that provides broad serotype coverage, induces mucosal and systemic immunity, and hinders primary intranasal colonization as well as invasive disease is highly desirable [6, 80]. Protein antigens conserved across pneumococcal serotypes may offer an alternative to serotype-restricted vaccination strategies [81–83].

As regards the effects on colonization, proteins could play a fundamental role. Most pneumococcal isolates that have been studied exhibit phase variation between two types of morphology that are differentiated based on their opaque or transparent colony. During the initial stages of colonization, transparent variants expressing a thinner capsule along with other characteristics promoting binding to host tissues prevail over opaque variants [84]. In fact, once at the epithelial surface, the expression of a thick capsule seems to be disadvantageous for the pneumococcus, because of its inhibitory effect on adherence. Moreover, while anti-capsular antibodies fail to protect against already established NP colonization, antibodies to pneumococcal adhesins can partially block the initiation of colonization and reduce the pneumococcal burden in the nasopharynx. In the same direction, it has been discovered that while naturally acquired immunity against IPD is thought to be dependent on anti-capsular antibodies, nasopharyngeal colonization by Spn also induces antibodies to protein antigens that could be protective [85].

Unfortunately, pneumo protein vaccines have not yet demonstrated a great efficacy in humans, despite preclinical evaluation of many of these proteins has shown the efficacy of passive transfer of immune sera in preventing sepsis and fatal pneumonia in mice, often against multiple serotypes of pneumococci [82]. Nevertheless, proteins can be considered an added value to PCVs to overcome some of their limitations.

A new technology platform called the Multiple Antigen Presentation System (MAPS) from Affinivax has been used for the development of a vaccine combining 24 pneumococcal CPS and a fusion of two conserved pneumococcal proteins, with dual role of carrier and antigen. This platform takes advantage of the high affinity, noncovalent binding between biotin and rhizavidin [86], and combine the potentiality of pneumo proteins to the protection elicited by CPSs. Using the biotin-avidin system, protein epitopes remain intact increasing anti-protein T-cell response as well. Results from a Phase 1 trial [87] have recently been confirmed in a Phase 2 trial, where the vaccine candidate showed a similar or better IgG and OPA immune response for the 13 serotypes shared with Prevnar13.

This technology also suggests that a direct covalent linkage between the CPS and the carrier protein is not necessary to mount a T-dependent response, but rather copresentation of the two components. As long as the interaction between the CPS and the protein survives the endosomal environment, the resulting antigen should be able to recruit T-cell help. CPSs non covalently associated with a protein carrier both mounted on bacteria-sized latex beads [88] demonstrated to behave similarly to PCVs. An advantage of this is the use of pneumococcal surface protein A (PspA) as carrier.

To overcome the difficulties and costs of including many CPS types in a single vaccine, whole-cell vaccines have been explored as alternatives as well. The development of such vaccines is less straightforward due to safety reasons and the complex immune response mounted after immunization [89]. It will be a while before any of these alternatives become available for human use.

## Analytical challenges because many conjugates are combined in multivalent formulations

The increasing number of polysaccharide serotypes included in the new vaccines against pneumococcal infections, up to 24 as recently tested in Phase 1/2 by Affinivax [87]and Vaxcyte [69], has implied a huge challenge for vaccine manufacturers both in term of vaccine formulation as well as for their analytical testing for product release.

For testing of monovalent process intermediates (i.e., purified plain polysaccharides, polysaccharide-protein conjugate - drug substances) and multivalent vaccine formulations, commonly adjuvanted with Alum, (drug product) a list of methods has been developed. In Tables 2, 3 and 4 an example list of the analytical methods used for purified plain polysaccharides, drug substances and drug product are reported.

**Table 2:** Example of analytical panel used for testing of purified polysaccharides

Category	Attribute	Test
Product Characterization	Polysaccharide Identity	^1^ H NMR
	O-Acetyl Content	^1^ H NMR
	Monosaccharides	Wet chemical assay, HPAEC-PAD
	Molecular Size Distribution	HPSEC
Purity *Product Related Residuals*	Core Polysaccharide Content	^31^P or ^1^ H NMR
	Protein Content	Lowry
	Nucleic Acid Content	Spectrophotometry
	Endotoxin Content	Chromogenic Kinetic Method
Purity *Process Related Residuals*	Alcohol Content	Spectrophotometry
	Water Content	Karl-Fisher

**Table 3:** Example of analytical panel used for testing of monovalent drug substances

Category	Attribute	Test
Product Characterization	Polysaccharide Identity	Immunochemical
	Protein Identity	Immunochemical
	Total Saccharide Content	Immunochemical/Physicochem
	Total Protein Content	Lowry
	Saccharide/Protein Ratio	Calculation
	Free Saccharide Content	Immunochemical/Physicochem
	Free Protein Content	HPSEC
	Molecular Size Distribution	HPSEC
Purity	Endotoxin Content	Chromogenic Kinetic Method
	Sterility	Membrane filtration

**Table 4:** Example of analytical panel used for testing of multivalent drug product

Category	Attribute	Test
Product Characterization	Polysaccharide Identity	Immunochemical
	Aluminium Content	Titrimetry
Purity	Sterility	Membrane filtration

Some of the tests used so far might be replaced by advanced technologies to enhance the analytical control and simplify the analytical panel. For instance, NMR might be used also for polysaccharide and alcohol content determination in the purified polysaccharide intermediate.

While the applicability of the existing assays at the level of monovalent process intermediates (i.e., purified polysaccharides, drug substances) seems to be reasonable feasible, even if it requires new reagents (antibodies) for immune testing, for product characterization of multivalent drug product the presence of an increased number of antigens significantly enhances the assay complexity in term of interference, cross-reactivity etc. Multiplexing approaches are also needed to reduce the time and cost for each single assay.

##  Licensing strategy

The effectiveness of conjugate-based vaccines against IPD in different age populations has been assessed by comparing the immunogenicity of each serotype versus a previously licensed vaccine. For instance, PCV20 effectiveness against IPD and pneumonia was inferred from comparative immunogenicity to US-licensed pneumococcal vaccines (PCV13 and PPV23). Immune responses elicited by PCV20 and the control pneumococcal vaccines were measured by an OPA assay. OPA assays were used to measure functional antibodies to Spn.

In adults ≥ 60 years of age, immune responses to all 13 matched serotypes induced by PCV20 were noninferior to those of the serotypes elicited by PVC13 one month after vaccination.

Surprisingly, the immune responses to 6 out of the 7 additional serotypes induced by PCV20 were noninferior to the immune responses raised by the same serotypes in PPSV23 one month after vaccination. The response to serotype 8 did not reach the statistical noninferiority criterion by a small extent [30, 73].

Considering the similarities between PCV20 and PCV13 in terms of manufacture

and formulation, the effectiveness demonstrated for PCV13 in preventing vaccine-type pneumococcal IPD and CAP [90] is supportive of the likely effectiveness of PCV20, particularly for the 13 common serotypes. Nonetheless, a study in adults aged ≥ 65 years is planned to confirm the clinical benefit of PCV20 for the prevention of pneumonia caused by the seven new serotypes [30].

## Conclusion

Despite there are more than 100 Spn serotypes, most strains responsible for disease are covered by currently available PCVs. However, serotype replacement due to vaccination and regional differences in dominant serotypes necessitate the expansion of existing vaccines to include additional serotypes. The research is still very active aiming at reducing the cost of manufacture and increasing serotype coverage avoiding possible issues related to immune-interference and carrier epitope suppression associated with increased valency. Also, the high cost of vaccines against Spn represents a significant barrier to uptake. Advancements in the field of conjugate vaccines can help simplifying vaccine manufacturing and analytics despite complex composition of required vaccines, increase protection through the addition of conserved protein antigens in vaccines formulation and overcome some limitations related to specific Spn structures and chemistries (e.g., Spn-1 and Spn-5).

## Data Availability

Not applicable.

## Declarations

### Disclaimer

Synflorix is a trademark of the GSK group of companies. Prevnar and Prevnar13 are trademarks of Wyeth LLC. Pneumosil is a trademark of Serum Institute of India Private Limited.
